# Lack of association between the CALM1 core promoter polymorphism (-16C/T) and susceptibility to knee osteoarthritis in a Chinese Han population

**DOI:** 10.1186/1471-2350-9-91

**Published:** 2008-10-22

**Authors:** Dongquan Shi, Haijian Ni, Jin Dai, Jianghui Qin, Yong Xu, Lunqing Zhu, Chen Yao, Zhenxing Shao, Dongyang Chen, Zhihong Xu, Long Yi, Shiro Ikegawa, Qing Jiang

**Affiliations:** 1 The Center of Diagnosis and Treatment for Joint Disease, Drum Tower Hospital Affiliated to Medical School of Nanjing University, Nanjing 210008, Jiangsu, PR China; 2 Laboratory for Bone and Joint Diseases, Model Animal Research Center, Nanjing University, Nanjing 210061, Jiangsu, PR China; 3 Department of Pathology, Medical School of Nanjing University, PR China; 4 Laboratory for Bone and Joint Diseases, Center for Genomic Medicine, Riken, Tokyo 108-8639, Japan

## Abstract

**Background:**

*CALM1 *gene encodes calmodulin (CaM), an important and ubiquitous eukaryotic Ca^2+^-binding protein. Several studies have indicated that a deficient CaM function is likely to be involved in the pathogenesis of osteoarthritis (OA). Using a convincing genome-wide association study, a Japanese group has recently demonstrated a genetic association between the *CALM1 *core promoter polymorphism (-16C/T transition SNP, rs12885713) and OA susceptibility. However, the subsequent association studies failed to provide consistent results in OA patients of differently selected populations. The present study is to evaluate the association of the -16C/T polymorphism with knee OA in a Chinese Han population.

**Methods:**

A case-control association study was conducted. The polymorphism was genotyped in 183 patients who had primary symptomatic knee OA with radiographic confirmation and in 210 matched controls. Allelic and genotypic frequencies were compared between patients and control subjects.

**Results:**

No significant difference was detected in genotype or allele distribution between knee OA and control groups (all *P *> 0.05). The association was also negative even after stratification by sex. Furthermore, no association between the -16C/T SNP genotype and the clinical variables age, sex, BMI (body mass index) and K/L (Kellgren/Lawrence) score was observed in OA patients.

**Conclusion:**

The present study suggests that the CALM1 core promoter polymorphism -16C/T is not a risk factor for knee OA susceptibility in the Chinese Han population. Further studies are needed to give a global view of this polymorphism in pathogenesis of OA.

## Background

Osteoarthritis (OA) is a common musculoskeletal disease among the elderly, characterized by the degradation of articular cartilage and formation of abnormal bone (osteophyte). It is a multifactorial disorder in which aging, genetic, hormonal and mechanical factors are all major contributors to its onset and progression [1]. Results of family-based and candidate gene studies have demonstrated a clear genetic component, particularly for early-onset OA [2-4]. The identification of candidate genes for OA susceptibility has mainly focused on genes encoding collagens (particularly for type II collagen), for other structural proteins of the extracellular cartilage matrix(ECM), the vitamin D and estrogen receptor genes, and for bone and cartilage growth factors [5]. And it is expected that some genes which regulate the formation, degradation, and repair of articular cartilage and subchondral bone metabolism may determine the occurrence of osteoarthritis. However, the specific underlying genetic factors and mechanisms in the development of osteoarthritis need to be further researched.

Calmodulin 1 gene (*CALM1*) encodes calmodulin (CaM), a ubiquitous eukaryotic Ca^2+^-binding protein which regulates many Ca^2+^-dependent cellular events by interacting with a heterogeneous group of target proteins [6]. Several studies have indicated that calmodulin probably plays a crucial role in articular cartilage on at least three aspects: regulating articular chondrogenesis [7], maintaining the cartilage phenotype in response to mechanical stimuli in mature chondrocytes [8,9] and modulating adhesion of chondrocytes to extracellular matrix proteins during the cartilage repairing process [10]. Consequently, a deficiency of Ca^2+^-calmodulin signal may be involved in the pathogenesis of OA.

Through a genome-wide association study, Mototani *et al *[7] previously reported a functional single nucleotide polymorphism (SNP) (-16C/T rs12885713) in the core promoter region of the *CALM1 *gene associated with a markedly increased prevalence of hip OA in the Japanese population. Subsequent functional analyses revealed that the susceptibility -16T allele of this polymorphism decreased *CALM1 *transcription both in vitro and in vivo. In addition, higher levels of *CALM1 *expression in hip and knee OA cartilage than in normal were also observed [7].

Together, these findings implicated the candidacy of *CALM1 *as a susceptibility gene for OA. However, subsequent association studies [11,12] failed to provide consistent results in OA patients of differently selected populations. To clarify its global relevance, the association needs to be confirmed by independent studies in different ethnic groups. The objective of this study is to assess the genetic association of the -16C/T polymorphism in *CALM1 *with knee OA in a Chinese Han population.

## Methods

### Subjects

A total of 183 patients with primary knee OA (124 women and 59 men) were recruited consecutively at the Center of Diagnosis and Treatment for Joint Disease, Drum Tower Hospital, affiliated to the Medical School of Nanjing University and 210 healthy control subjects (142 women and 68 men) were recruited at the Center of Physical Examination. All subjects included in the study were Han Chinese living in or around Nanjing. The study was approved by the ethics review committee of the Medical School of Nanjing University and written informed consent was obtained from all participants. The ascertainment criteria for both OA patients and control subjects were according to the previously published study by Jiang *et al *[13]. In the study, other etiologies causing knee diseases such as inflammatory arthritis (rheumatoid, polyarthritic, or autoimmune disease), posttraumatic or post-septic arthritis, skeletal dysplasia or developmental dysplasia were excluded. Radiographic OA was assessed using the Kellgren/Lawrence (K/L) grading system [14]. Only patients with K/L grades of 2 or higher were included. All the control never had any signs or symptoms of arthritis or joint diseases (pain, swelling, tenderness, or restriction of movement)

### Genotyping

Genomic DNA was extracted from peripheral blood leukocytes using the Chelex-100 method [15] or from buccal swabs using the DNA IQ System (Promega, Madison, WI) according to the manufacturer's instructions. PCR amplifications were performed for the -16C/T SNP using forward primer 5'GCG GGA GGC CTG GCG AAC3' and reverse primer 5'CAC TGC CGG AGC GCG AGT ***G***T3'. The italic and boldfaced nucleotide in the reverse primer is a G as opposed to the C that exists in the *CALM1 *sequence in order to create a APAL‡T restriction enzyme site in the T-allele of the SNP which is absent in the C-allele. The amplification solution system was supplemented with 5% dimethyl sulfoxide (DMSO) [11]. Amplification was carried out as follows: 31 cycles consisting of 1 min of denaturation at 94°C, 1 min of annealing at 62°C and 1 min of extension at 72°C with an initial denaturation step of 2 min at 96°C and a final extension of 15 min at 72°C. The 148 bp PCR product was then digested with APALI (New England Biolabs, Hitchin, UK). After that, a T-allele was digested into two fragments, of 125 bp and 23 bp, whilst a C-allele remained uncut. Restriction fragments were separated by 3% agarose gel electrophoresis and ethidium bromide staining. To verify the results of the original assay, randomly selected 10% samples were amplified using a second pair of sequencing primers: forward 5'CTT CCC CGA CCC AGC GTA3' and reverse 5'CGC CGC CTG ACT ACG AGT AAC3'. The 244 bp product was then digested with HpyCH4V. A T-allele was cut into two fragments, of 174 bp and 70 bp, while a C-allele, again, remained uncut. Genotyping was done by laboratory personnel blinded to case status, and a random 5% of the samples were repeated to validate genotyping procedures. Two authors independently reviewed the genotyping results, data entry, and statistical analyses.

### Statistical analysis

For this case-control association study, we used standard χ^2 ^tests to determine the significance of differences in allelic and genotypic frequencies between OA patients and control subjects. P < 0.05 was considered statistically significant. Allele and genotype proportions were tested for Hardy-Weinberg equilibrium. Clinical variables age, sex, BMI (body mass index) and K/L score of OA patients with different genotype were compared using χ^2 ^tests or Kruskal-Wallis tests. Power estimates were calculated using the software R.

## Results

Table 1 shows the basic characteristics of the study population. The patients and the controls were matched in age and sex. Over 59% of the patients had a K/L score of 3 or 4. Table 2 shows the allele and genotype distributions for cases and controls. Both of the allele and genotype proportions were in Hardy-Weinberg equilibrium. The recessive allele (T) and genotype (TT) frequencies of controls (20.5% and 3.8%, respectively) were similar to those previously published in the Japan population [7] (*P *= 0.023 and 0.064) but not to those of UK Caucasians [11] (*P *= 0.000 and 0.000). In the association study, no significant differences between cases and controls were observed either in allele (*P *= 0.996) or genotype (*P *= 0.802) frequencies, and the allele frequencies were even the same between the two groups. Stratifying the samples by sex did not result in any significant association (Table 2). Moreover, we did not find any association between the -16C/T SNP genotype and the clinical variables age, sex, BMI and K/L score in OA patients (Table 3 and Table 4). The power for this study was over 0.05.

**Table 1:** Subject characteristics

Subjects	Number	Sex		Age (years)		BMI		K-L score	
					
		Female(%)	Male	Mean	SD(±)	Mean	SD(±)	=2	≥ 3(%)
OA cases	183	124(67.8)	59	58.6	13.5	25.4	3.5	75	108(59)
Controls	210	142(67.6)	68	57.7	11.7	23.2	3.9		

BMI: body mass index.

**Table 2:** Association of SNP rs12885713 between OA cases and controls

Subjects	Allele			Genotype			
				
	C(%)	T(%)	*P *value	CC(%)	CT(%)	TT(%)	*P *value*
All cases (n = 183)	291(79.5)	75(20.5)	0.996	117(63.9)	57(31.1)	9(4.9)	0.802
All controls (n = 210)	334(79.5)	86(20.5)		132(62.9)	70(33.3)	8(3.8)	
							
Female cases (n = 124)	198(79.8)	50(20.2)	0.818	79(63.7)	40(32.3)	5(4.0)	0.941
Female controls (n = 142)	229(80.6)	55(19.4)		93(65.5)	43(30.3)	6(4.2)	
							
Male cases (n = 59)	93(78.8)	25(21.2)	0.758	38(64.4)	17(28.8)	4(6.8)	0.313
Male controls (n = 68)	105(77.2)	31(22.8)		39(57.4)	27(39.7)	2(2.9)	

*Odds ratio and *P *value were calculated on recessive model (TT versus CT + CC).

**Table 3:** Clinical variables in the OA patients and the rs12885713 genotype

Variable	Genotype						
		
	CC		CT		TT		*P *value
				
	Mean	SD	Mean	SD	Mean	SD	
Age	57.7	13.0	59.6	14.7	64.4	11.3	0.23^a^
BMI	25.4	3.1	25.7	4.2	24.7	4.1	0.87^a^
Sex (Male %)	32.5		29.8		44.4		0.68^b^

BMI: body mass index.

a The *P *values were calculated using Kruskal-Wallis test.

b The *P *values were calculated using χ^2 ^test.

**Table 4:** Genotype distribution of OA subgroups stratified by K/L score

		Genotype			
			
K/L score	Total	CC(%)	CT(%)	TT(%)	*P *value*
2	75	50(66.7)	21(28.0)	4(5.3)	0.86
3	54	37(68.5)	15(27.8)	2(3.7)	
4	54	32(59.3)	19(35.2)	3(5.5)	

* The *P *value was calculated using 3 × 3 χ^2 ^test.

## Discussion

Through a large-scale association study with subsequent functional researches, the Japanese group have demonstrated a convincing association between the *CALM1 *core promoter polymorphism and Japanese hip OA. The well clarified role Ca^2+^-calmodulin signal played in chondrogenesis and normal cartilage phenotype maintaining, together with the results of the microarray analysis [7] showing higher levels of *CALM1 *expression both in hip and knee OA, have interested us in assessing whether this genetic polymorphism is also associated with OA in a Chinese Han population. However, we failed to find any positive result. Before the present study, this potential association had also been studied in UK Caucasian populations [11,12], yielding no positive findings either in hip or knee OA patients. Together, these studies highlight the complexity and diversity of genetic contributions to such a multifactorial disease as OA.

The "T" allele and "TT" genotype frequencies of controls in present study were very similar to those in the original study of the Japan group but different to those in the UK study [12]. The "T" allele even seemed to be the major allele in UK Caucasians instead of the allele "C" which was dominant in both the present and the Japanese studies, indicating a heterogenous nature of this polymorphism between Asians and UK Caucasians. The different ethnic background and the potential linkage disequilibrium (LD) of the -16C/T locus with some other more relevant allele or alleles within the CALM1 gene that differ in different populations, may be one of the most likely explanations for the failure to replicate the association of -16C/T with OA susceptibility in UK Caucasians.

Recently, several population-based association studies for other OA susceptibility genes have shown consistent results in the Japanese and Chinese Han populations [13,16]. Populations from neighboring regions may have greater similarities in genome for the fact that they share more recent common ancestors. Therefore, their allele frequencies are more highly correlated, a pattern that is commonly manifested as a cline of allele frequencies [17]. All these together had encouraged us to expect a positive association of -16C/T polymorphism in our population-based study, except for the fact that there is an obvious difference in clinical phenotype of OA patients between the present and the original Japanese studies -the different joint site affected, which may account for the inconsistent results of the two studies. It is becoming more apparent that the nature of OA genetic susceptibility is likely to vary between different joint sites [18]. In the RHOB and TXNDC3 studies, different associations were detected for the different affected joint sites [19-21]. A relatively small population size might have prevented from seeing the association. The proportion of genetic contribution of certain polymorphic locus to OA susceptibility may be influenced by other local environmental factors such as anatomical and biomechanical effects, and by some joint-specific genetic factors most of which were postulated to be involved in cell signalling and signal transduction [18,22]. Interestingly, *CALM1 *is only one member of the *CALM *family by which mammalian CaM is encoded. The *CALM *family includes three nonallelic genes *CALM1*, *CALM2 *and *CALM3 *located on chromosomes 14q24-q31, 2p21.1-p21.3 and 19q13.2-q13.3, respectively, but all these three genes encode an identical 148 amino acid protein CaM [23]. However, the transcriptional activity and the proportion of each gene contributing to the local mRNA pools seem to differ during cell differentiation processes or in different biochemical microenvironments [24,25]. Thus, the other two *CALM *genes that are more functionally active in the knee joint might have decreased the extent to which the *CALM1 *OA risk is manifest. To make our study more complete and comparable, it is necessary to collect enough samples of hip OA despite of the very much lower prevalence [26], and assess the association between this polymorphism and hip OA in the same Chinese population.

Moreover, Hoaglund [27] has recently made a comment on the ascertainment criteria used for the patient enrollment in the Japanese association study and has suggested that the *CALM1 *core promoter polymorphism may be associated with Japanese congenital hip disease causing secondary hip OA rather than primary hip OA. Colleagues in our department are now trying to identify this hypothesis by assessing the association in patients of developmental dysplasia of the hip (DDH) which may represent a severe phenotype of congenital hip disease.

## Conclusion

The present study did not find any significant association between the *CALM1 *core promoter polymorphism (-16C/T) and susceptibility to knee OA in the Chinese Han population. Further studies are necessary to identify the association of this polymorphism with hip OA in our population and with OA in other different populations on different joint sites to give a global view of this polymorphism in pathogenesis of OA.

## Competing interests

The authors declare that they have no competing interests.

## Authors' contributions

HN carried out the molecular genetic studies, performed the statistical analysis and drafted the manuscript. JD, JQ, YX, CY, LZ participated in sample collection and DNA extraction. LY participated in the design of the study and help to carry out the molecular genetic studies. QJ and DS conceived of the study, and participated in its design and coordination and helped to draft the manuscript. All authors read and approved the final manuscript.

## Pre-publication history

The pre-publication history for this paper can be accessed here:


